# Moral Courage as a Predictor of Moral Comfort in Critical Care Nurses

**DOI:** 10.1155/jonm/2257642

**Published:** 2025-12-25

**Authors:** Mansoureh Zagheri-Tafreshi, Sara Etemadi, Parvin Babaei, Maasoumeh Barkhordari-Sharifabad

**Affiliations:** ^1^ School of Nursing and Midwifery, Shahid Beheshti University of Medical Sciences, Tehran, Tehran Province, Iran, sbmu.ac.ir; ^2^ Department of Nursing, YMS. C., Islamic Azad University, Yazd, Yazd Province, Iran, azad.ac.ir; ^3^ Shahid Rahnemoun Hospital, Shahid Sadoughi University of Medical Sciences, Yazd, Yazd Province, Iran, ssu.ac.ir; ^4^ Rafsanjan University of Medical Sciences, Rafsanjan, Kerman Province, Iran, rums.ac.ir

**Keywords:** ICU, moral comfort, moral courage, moral distress, nurse

## Abstract

**Background:**

Improving the experience of moral comfort is essential for helping intensive care unit (ICU) nurses achieve peace of mind while also ensuring the safety and comfort of their patients. Moral comfort is promoted through the presence of both personal and environmental factors, among which moral courage is a key personal element.

**Aim:**

The aim of this study is to find out the role of moral courage in the moral comfort of nurses working in ICUs.

**Design:**

This study used a cross‐sectional design.

**Methods:**

This study involved 153 nurses working in the ICUs of two hospitals affiliated with Shahid Sadoughi University of Medical Sciences, Yazd, Iran, between April 2025 and June 2025. Data were collected using a demographic form and two questionnaires on moral comfort and moral courage. Data were analyzed using descriptive and analytical statistics in SPSS22.

**Results:**

The mean scores for moral courage and moral comfort were 83.66 ± 13.17 and 99.86 ± 16.27, respectively. There was a statistically significant positive correlation between moral courage and moral comfort (*r* = 0.595, *p* < 0.001). Furthermore, moral courage predicted 35% variance in moral comfort.

**Conclusion:**

The findings indicate that moral courage is a significant predictor of moral comfort among ICU nurses. Nurses with higher levels of moral courage are more likely to experience moral comfort, which may enhance their psychological well‐being and professional performance in ethically challenging environments such as the ICU.

**Implications for Nursing Management:**

Nursing managers should recognize the crucial role of moral courage in fostering moral comfort and strive to create supportive environments that empower nurses to act in alignment with their ethical values. To enhance moral comfort, healthcare organizations should invest in cultivating moral courage through targeted training programs, supportive leadership, and a culture that encourages nurses to voice concerns and advocate for their patients.

## 1. Introduction

Intensive care units (ICUs) are highly stressful and complex hospital settings [1, 2], with mortality rates ranging from 20% to 48.7% depending on patients’ conditions [3, 4]. ICU nurses face heavy workloads, complex ethical decisions, and responsibility for both patient safety and emotional well‐being [5]. In Iran, these challenges are compounded by rigid hierarchical relationships between physicians and nurses, lack of formal ethics committees, absence of clear end‐of‐life care guidelines, and severe staffing shortages [6]. Such challenges can lead to moral distress [7], defined as the experience when nurses recognize the ethically appropriate action but cannot perform it due to constraints [8]. Moral distress may result in a range of negative emotional and professional outcomes, including emotional exhaustion, reduced empathy, feelings of helplessness, and impaired quality of care [9–11].

While the absence of moral distress indicates that nurses do not experience the negative psychological impacts, it does not necessarily imply that they experience comfort or a positive psychological state [12]. Kolcaba conceptualizes comfort as a holistic, multidimensional experience encompassing physical, psychospiritual, sociocultural, and environmental aspects, achieved through fulfillment of needs for relief, ease, and transcendence [13]. Within this theoretical framework, moral comfort can be understood as a form of holistic ease that arises when ethical beliefs and professional actions are congruent, representing a positive moral state that extends beyond the mere absence of moral distress [14, 15].

Moral comfort, first introduced by Wurzbach​ in 1996 [16], is an active, positive state in which nurses experience psychological well‐being and moral fulfillment [14]. It manifests in nurses’ professional practice as a sense of internal satisfaction and confidence when making ethically sound decisions [16]. The experience of moral comfort is essential for maintaining integrated moral and emotional well‐being [17]. It contributes to nurses’ emotional stability and job satisfaction, reduces burnout, strengthens commitment to patient advocacy [18], and ultimately enhances patient safety, quality of care, and family well‐being. Despite its benefits, moral comfort has received less attention than moral distress [19–21].

Moral comfort is enhanced by certain personal and environmental factors [19, 20], with moral courage being one of the personal factors. Moral courage is defined as the ability to logically defend moral principles and act according to them despite potential unfavorable consequences [22], such as humiliation, bans, contempt, job loss, or loss of social status [23]. It can facilitate the fulfillment of nurses’ psychospiritual and transcendence needs, as conceptualized in Kolcaba’s comfort theory [13]. Moral courage serves as a powerful approach to confronting moral issues [24], liberates nurses from experiencing moral distress [25, 26], and helps them attain a sense of internal calmness and professional integration [27]. However, findings from some studies [28–30] suggest that nurses with moral courage may still suffer from moral distress due to a lack of sufficient power to achieve their goals. Moral courage contributes to providing safe and professional patient care [31–33], to being more attentive to others’ discomfort, and to fostering a greater sense of empathy and kindness [34].

The positive potential of moral comfort in enhancing personal and professional outcomes underscores the necessity for studies focusing on this concept [20], even though research in this field is currently limited. Given the specific challenges faced by nurses in Iranian hospitals—including shortages of equipment and personnel, inadequate support systems, and limitations in creating cultural and educational environments conducive to the development of professional ethics [35]—these factors may increase the risk of moral distress among nurses. It appears that promoting moral courage, as a key personal resource, can help nurses confront these challenges more effectively and thereby enhance their moral comfort. Moral comfort is essential to support nurses’ psychological well‐being, professional integration, and the delivery of high‐quality patient care. However, moral comfort remains underexplored within the Iranian nursing profession. Accordingly, this study was designed to determine the role of moral courage in nurses’ moral comfort among those working in ICUs in Iran.

## 2. Materials and Methods

### 2.1. Study Design

This research is a cross‐sectional study.

### 2.2. Participation

The study population consisted of nurses working in the ICUs of Shahid Rahnamoun and Shahid Sadoughi hospitals, affiliated with Shahid Sadoughi University of Medical Sciences, Yazd, Iran, between April 2025 and June 2025. The sample size was determined to be 160, calculated based on a 95% confidence level, 80% test power, and a correlation coefficient of 0.23 obtained from a pilot study involving 30 nurses and an additional 10% for potential attrition. Inclusion criteria were holding a Bachelor of Science in Nursing and a higher degree and having more than 6 months of experience in ICUs. Exclusion criteria were being on sick leave or paid leave during the study period. Sampling was conducted using a simple random method. First, a list of eligible nurses working in the ICUs was obtained from the hospitals’ nursing registries. Each nurse on the list was assigned a unique number, and 160 nurses were randomly selected using computer‐generated random numbers. After identifying the samples, all participants were informed about the study’s purpose and procedures. Upon obtaining their informed consent, the researchers personally distributed the questionnaires to nurses at the beginning of their shifts. Participants were asked to complete them individually at a convenient time and were provided with the researchers’ contact information for any questions or clarifications. The researchers then collected the completed questionnaires at the end of the same shift.

### 2.3. Data Collection

A questionnaire with three different sections was used for data gathering:

A demographic information form consisted of age, gender, education level, marital status, work shift, type of employment, years of clinical experience, ICUs’ experience, and participation in ethics workshops.

The Moral Courage Questionnaire was developed by Sekerka et al. in 2009 [36]. This questionnaire consists of 15 items in five dimensions: moral agency, multiple values, threat tolerance, going beyond compliance, and moral goals. Each dimension has three separate items. Responses are rated on a 7‐point Likert scale ranging from 1 “never true” to 7 “always true”. Each dimension can score between 3 and 21, and the total score ranges from 15 to 105, with higher scores indicating higher moral courage [36]. The validity of the Persian version was confirmed in a study by Mohammadi et al. with a validity rate of 81%, and its reliability was assessed by Cronbach’s alpha (*α* = 0.85) [29]. In this study, the moral courage Questionnaire demonstrated excellent internal consistency for the total score (Cronbach’s *α* = 0.93). The reliability of the five subscales was as follows: moral agency (*α* = 0.75), multiple values (*α* = 0.77), threat tolerance (*α* = 0.84), going beyond compliance (*α* = 0.82), and moral goals (*α* = 0.79).

The Moral Comfort Questionnaire (MCQ) was first developed by Bermudez in 2020 [21]. There are 35 items divided into two parts: the first one relates to a specific situation, and the second part evaluates the nurse’s general experience. In the first part with 15 items, respondents are asked to recall a specific moral dilemma faced in the past six months. For the second part, the participants reflect on their general performance by answering 20 items. Each item is rated on a 4‐point Likert scale as 4 “strongly agree”, 3 “somewhat agree”, 2 “somewhat disagree”, 1 “strongly disagree”. This questionnaire has two dimensions: 21 items assess morally‐related individual factors, and the other dimension with 14 items assesses environment‐related external factors. The total score of moral comfort ranges from 35 to 140, with higher scores indicating greater moral comfort [21]. The face, content, and construct validity have been confirmed by Abbasivand and Barkhordari‐Sharifabad, and its reliability was reported as *α* = 0.888, with an interclass correlation coefficient of *r* = 0.825, indicating good reliability [14]. In the present study, the MCQ demonstrated excellent reliability (*α* = 0.94), with both the individual and environmental factors subscales showing high internal consistency (*α* = 0.92 and 0.89, respectively).

### 2.4. Ethical Considerations

The ethics committee approved the submitted proposal (IR.IAU.KHUISF.REC.1404.008). Ethical considerations carefully observed in this study included obtaining informed consent, providing information to participants about the research objectives and methods, ensuring voluntary participation, and maintaining the confidentiality of participant information.

### 2.5. Data Analysis

Any missing data were examined prior to analysis. Cases with incomplete questionnaires were excluded from the analysis (listwise deletion). Data analysis was performed using SPSS 22. Descriptive statistics (mean, standard deviation, and frequency) were calculated to summarize participants’ characteristics. The Kolmogorov–Smirnov test was used to assess the normality of the data distribution (*p* > 0.05). Pearson correlation coefficients were used to examine bivariate relationships between continuous variables, and independent *t*‐tests and one‐way ANOVA were applied to compare moral comfort and moral courage across participants’ demographic characteristics. A linear regression was first conducted to examine the predictive effect of overall moral courage on moral comfort. Subsequently, theory‐driven hierarchical regression analyses were conducted to assess the predictive effects of the five dimensions of moral courage on moral comfort. The entry order of the five dimensions in the hierarchical regression was based on the theoretical themes and conceptual ordering provided by the original developer of the Moral Courage Scale: with moral agency as the foundational dimension, representing the individual’s capacity to act according to ethical principles, which enables the effective expression of all other dimensions, followed by multiple values, threat tolerance, going beyond compliance, and moral goals [36]. Before conducting these regression analyses, all assumptions (linearity, normality, homoscedasticity, and absence of multicollinearity) were examined.

## 3. Results

A total of 153 participants completed the questionnaires (response rate = 95.62%). The average age of the nurses was 36.25 ± 8.07. The mean clinical work experience was 11.76 ± 7.66 years, and the mean work experience in the ICUs was 81.84 ± 71.64 months. Most participants were female (57.5%), married (66.0%), and holding a BSN degree (85.0%). Most nurses were formally employed (56.9%), and 59.5% had attended an ethics workshop previously (Table 1).

**Table 1:** Distribution of sociodemographic characteristics of nurses.

Variable	*N* (%)	Mean ± SD
Age		36.25 ± 8.07
Clinical work experience (years)		11.76 ± 7.66
Work experience in ICU (months)		81.84 ± 71.64
Gender		
Male	65 (42.5)	
Female	88 (57.5)	
Marital status		
Married	101 (66.0)	
Single	52 (34.0)	
Level of education		
BS.	130 (85.0)	
M.Sc. and higher	23 (15.0)	
Work shift		
Fixed	10 (6.5)	
Rotational	143 (93.5)	
Employment status		
Formal	87 (56.9)	
Contractual	40 (26.1)	
Contract	6 (3.9)	
Obligatory service	20 (13.1)	
Participate in an ethics workshop		
Yes	91 (59.5)	
No	62 (40.5)	

The mean scores for moral courage and moral comfort were 83.66 ± 13.17 and 99.86 ± 16.27, respectively. Among the dimensions of moral courage, the highest mean score was related to the “moral goals” (17.04 ± 2.79), and the lowest mean score belonged to the “multiple values” (16.19 ± 3.09) (Table 2).

**Table 2:** Descriptive findings of moral courage and moral comfort.

Variables	Number of items	Min	Max	Mean ± SD
Moral agency	3	5.00	21.00	16.97 ± 3.18
Multiple values	3	5.00	21.00	16.19 ± 3.09
Threat tolerance	3	6.00	21.00	16.42 ± 3.46
Going beyond compliance	3	5.00	21.00	17.03 ± 2.88
Moral goals	3	7.00	21.00	17.04 ± 2.79
Moral courage (total)	15	41.00	105.00	83.66 ± 13.17

Morally‐related individual factors	21	33.00	83.00	63.57 ± 10.17
Environment‐related external factors	14	17.00	56.00	36.29 ± 7.90
Moral comfort (total)	35	52.00	139.00	99.86 ± 16.27

*Note:* Min: minimum; Max: maximum.

Abbreviation: SD, standard deviation.

Considering unequal item numbers in the two dimensions of moral comfort and weighing each dimension, the scores were converted to 100 before comparing them. The mean standardized score for the “morally‐related individual factors” dimension was 67.58 ± 16.15, and for the “environment‐related external factors” dimension was 53.06 ± 18.81, which means participants scored higher on the “morally‐related individual factors” dimension.

The Kolmogorov–Smirnov test was used to assess the normality of continuous variables. As shown in Table 3, all variables, including moral courage, moral comfort, and demographic factors, had *p* > 0.05, indicating normal distributions. Therefore, the use of parametric statistical tests, such as Pearson correlation and regression analyses, was considered appropriate.

**Table 3:** Results of Kolmogorov–Smirnov test.

Variables	K‐S statistic	*p* value
Moral agency	0.052	0.071
Multiple values	0.071	0.065
Threat tolerance	0.060	0.056
Going beyond compliance	0.070	0.064
Moral goals	0.050	0.071
Moral courage (total)	0.060	0.057

Morally‐related individual factors	0.065	0.058
Environment‐related external factors	0.070	0.064
Moral comfort (total)	0.043	0.200

Age	0.063	0.054
Clinical work experience (years)	0.062	0.051
Work experience in ICU (months)	0.068	0.062

Pearson correlation analyses indicated statistically significant positive correlations between moral courage and its dimensions with moral comfort and its dimensions (*p* < 0.01) (Table 4).

**Table 4:** Correlation matrix of moral courage and moral comfort.

Variables	1	2	3	4	5	6	7	8	9
1‐Moral agency	1								
2‐Multiple values	0.566^∗∗^	1							
3‐Threat tolerance	0.664^∗∗^	0.577^∗∗^	1						
4‐Going beyond compliance	0.652^∗∗^	0.707^∗∗^	0.790^∗∗^	1					
5‐Moral goals	0.585^∗∗^	0.617^∗∗^	0.712^∗∗^	0.781^∗∗^	1				
6‐Moral courage (total)	0.815^∗∗^	0.808^∗∗^	0.882^∗∗^	0.915^∗∗^	0.856^∗∗^	1			

7‐Morally‐related individual factors	0.508^∗∗^	0.419^∗∗^	0.526^∗∗^	0.587^∗∗^	0.534^∗∗^	0.601^∗∗^	1		
8‐Environment‐related external factors	0.387^∗∗^	0.357^∗∗^	0.401^∗∗^	0.387^∗∗^	0.404^∗∗^	0.453^∗∗^	0.615^∗∗^	1	
9‐Moral comfort (total)	0.505^∗∗^	0.435^∗∗^	0.524^∗∗^	0.555^∗∗^	0.530^∗∗^	0.595^∗∗^	0.924^∗∗^	0.870^∗∗^	1

^∗∗^Correlation is significant at the 0.01 level (2‐tailed).

Potential confounding variables, including age, gender, clinical work experience, ICU work experience, education level, marital status, work shift, type of employment, and prior participation in ethics workshops, were examined for their associations with moral courage and moral comfort. Results showed that moral courage was significantly and positively correlated with only age (*r* = 0.16, *p* = 0.04), clinical work experience (*r* = 0.16, *p* = 0.04), and ICU work experience (*r* = 0.19, *p* = 0.01). No significant correlations were observed between moral courage and the other demographic variables (gender, education level, marital status, work shift, type of employment, and participation in ethics workshops), nor between moral comfort and any demographic variables (*p* > 0.05).

Regression analysis was used to examine the role of moral courage in predicting nurses’ moral comfort. It should be noted that, since no significant associations were found between demographic variables and moral comfort, these variables were not included in the regression model. Multicollinearity was assessed using the variance inflation factor (VIF) and tolerance statistics. Both VIF and tolerance values were 1.000, confirming the absence of multicollinearity in the model. A scatterplot of moral courage (predictor) against moral comfort (outcome) showed an approximately linear relationship, supporting the linearity assumption for regression analysis. Homoscedasticity was assessed by plotting standardized residuals against standardized predicted values. Residuals were evenly distributed around zero, showing no discernible pattern, indicating that the assumption of homoscedasticity was met. Linear regression analysis indicated that the correlation coefficient (R) between moral comfort (as the dependent variable) and moral courage (as the predictor variable) was 0.595, and the coefficient of determination (*R* ^2^) was 0.350. The ANOVA results showed that the *R* ^2^ value was statistically significant (*p* < 0.001, *F* [1, 151] = 82.948) (Table 5).

**Table 5:** Linear regression analysis for moral comfort.

Predictor	Unstandardized coefficients	Standardized coefficients	*t*	*p*	95% confidence interval	
	B	Beta			Lower	Upper
(Constant)	38.337		5.606	< 0.001	24.83	51.85
Moral courage	0.735	0.595	9.108	< 0.001	0.58	0.89

*Note: F* [1, 151] = 82.948; *p* < 0.001; *R* = 0.595; adjusted *R* ^2^ = 0.350. Dependent variable: moral comfort.

A theory‐driven hierarchical regression analysis was conducted to examine the predictive power of the five dimensions of moral courage on nurses’ moral comfort. Before conducting the regression, all assumptions (linearity, normality, homoscedasticity, and absence of multicollinearity) were examined and found to be satisfactorily met.

Table 6 presents the model summary. ANOVA confirmed that all hierarchical regression models were statistically significant (*p* < 0.001). In the first step, moral agency alone accounted for 25.1% of the variance in moral comfort (adjusted *R* ^2^ = 0.251, *p* < 0.001). Adding multiple values in the second step significantly improved the model (adjusted *R* ^2^ = 0.279, *p* < 0.01). In the third step, threat tolerance was entered and emerged as a significant predictor (*β* = 0.288, *p* = 0.003), while the contribution of multiple values was no longer significant; the model explained 31.6% of the variance. The fourth model included going beyond compliance, and this dimension remained a significant predictor (*β* = 0.280, *p* = 0.028), further increasing the explained variance to 33.4%. Finally, moral goals were added in the fifth model; however, this dimension was not significant (*p* = 0.101), and the model explained 34.1% of the variance in moral comfort (Table 6).

**Table 6:** Summary of theory‐driven hierarchical regression models predicting nurses’ moral comfort.

Model	Beta	*T*	*p*	95% CI		*F*	*p*	*R*	*R* ^2^	Adjusted *R* ^2^
				Lower	Upper					
1						51.823	< 0.001	0.505	0.256	0.251
(Constant)		9.038	0.000	43.765	68.254					
Moral agency	0.505	7.199	0.000	1.874	3.293					
2						30.369	< 0.001	0.537	0.288	0.279
(Constant)		7.096	0.000	34.722	61.521					
Moral agency	0.381	4.564	0.000	1.105	2.793					
Multiple values	0.219	2.625	0.010	0.285	2.020					
3						24.433	< 0.001	0.574	0.330	0.316
(Constant)		6.840	0.000	32.383	58.694					
Moral agency	0.238	2.535	0.012	0.269	2.167					
Multiple values	0.134	1.558	0.121	−0.189	1.598					
Threat tolerance	0.288	3.038	0.003	0.474	2.235					
4						20.039	< 0.001	0.593	0.351	0.334
(Constant)		5.887	0.000	27.060	54.406					
Moral agency	0.206	2.189	0.030	0.102	2.000					
Multiple values	0.038	0.393	0.695	−0.794	1.188					
Threat tolerance	0.144	1.266	0.208	−0.380	1.736					
Going beyond compliance	0.280	2.219	0.028	0.173	2.993					
5						16.760	< 0.001	0.603	0.363	0.341
(Constant)		5.090	0.000	22.591	51.270					
Moral agency	0.196	2.098	0.038	0.058	1.948					
Multiple values	0.017	0.181	0.857	−0.902	1.084					
Threat tolerance	0.103	0.888	0.376	−0.593	1.561					
Going beyond compliance	0.192	1.402	0.163	−0.443	2.607					
Moral goals	0.181	1.648	0.101	−0.211	2.329					

*Note:* Dependent variable: moral comfort.

Overall, moral agency emerged as the most consistent and robust predictor across all models. Threat tolerance and going beyond compliance also made meaningful contributions, whereas multiple values and moral goals were not significant in the final model. The full model accounted for approximately 34% of the variance in moral comfort (Table 6).

## 4. Discussion

This study was conducted to determine the role of moral courage in the moral comfort of nurses working in ICUs. The findings showed that moral comfort improves with higher levels of moral courage and that moral courage predicts 35% of the variance in moral comfort. These results are consistent with those of other studies [19, 20, 25].

According to the findings of Corley and Minick, moral courage generates motivation for moral action or agency, which is one of the internal factors influencing moral comfort [19]. Bermudez suggested that the willingness to take moral action despite personal consequences is a characteristic of moral comfort that requires moral courage [20]. Similarly, the findings of Savel and Munro [25], as well as Karampourian et al. [26], suggest that nurses may not experience moral distress (the opposite of moral comfort) if they possess sufficient moral courage. However, the findings of some studies indicate a positive correlation between moral courage and moral distress [28–30], suggesting that nurses with high moral courage may make brave decisions when faced with ethical challenges, but due to a lack of sufficient authority, they may fail to achieve their goals, which leads to moral distress. Such differences in findings may reflect distinctions between the concept of moral comfort and the mere absence of moral distress, in addition to the variations in research methods, such as measurement tools. Moral courage can lead to moral comfort only if individuals perceive their actions as resulting in positive moral outcomes. Although Corley believes that personal factors, such as moral courage, contribute to moral comfort, and that their absence leads to moral distress [19], further research on this topic is needed.

A tentative conceptual model can be outlined for the interrelationship between these variables: moral courage enhances moral comfort directly, and it may also contribute indirectly by reducing moral distress, which in turn undermines moral comfort. This framework highlights the need for future studies to clarify how these dynamics unfold across different nursing contexts.

Findings of this study revealed that among the five dimensions of moral courage, “moral agency”—defined as the nurses’ readiness to assume responsibility and act according to ethical principles—was the strongest predictor of moral comfort. When nurses are able to exercise moral agency, they take ownership of ethical challenges and make decisions that align with professional standards and patient‐centered values. According to Milliken [37], moral agency involves the capacity to act ethically while re‐centering decision‐making on patient values, allowing nurses to navigate complex situations without compromising their ethical integrity. In this context, the active engagement in morally responsible actions provides nurses with a sense of satisfaction, coherence, and ethical reassurance in their practice, thereby increasing their moral comfort. These findings suggest that promoting moral agency in clinical settings can be an effective strategy to enhance nurses’ moral comfort and support their professional development.

The results showed that the mean score of moral comfort among nurses working in ICUs was 99.86 ± 16.27 on a scale of 35–140. This score is lower than that reported in a previous study in Iran (112.75 ± 13.18) [14], which may be due to differences in the study population and the participants’ individual characteristics [14, 38]. In international studies, moral comfort among nurses in the USA has been reported as 123.04 [21]. This difference may reflect variations in healthcare systems, nursing roles, and cultural factors that shape professional ethics and moral experiences in nursing practice. At the organizational level, elements such as hierarchy, regulations, support, workload, culture, and resources (staff and equipment) have been identified as factors influencing nurses’ professional moral comfort [39]. Nevertheless, studies that specifically evaluate the level of moral comfort are very limited. Some research has explored nurses’ perceptions of comfort related to different aspects of care. For instance, in some studies, a high level of perceived comfort regarding moral issues has been reported among oncology nurses [40], while a moderate level of perceived comfort has been observed among nurses working in emergency departments [41]. Identifying moral comfort and understanding how to promote it is particularly important, as it can facilitate ethical decision‐making and moral action among nurses [14]. Enhancing moral comfort not only supports nurses’ professional development but also contributes to the quality of patient care, patient safety, and positive patient outcomes [21].

In the present study, the findings reveal that individual factors contribute more significantly to nurses’ moral comfort than the environmental factors, which is consistent with other studies [14, 21]. Although environmental factors—such as organizational support, institutional policies, and the ethical climate of ICUs—clearly influence nurses’ moral comfort, findings of this study show that the impact of these external factors may be less significant than that of individual characteristics. Nurses may face various organizational barriers, such as staffing shortage, limited resources, or conflicting policies, which can make moral action difficult despite organizational efforts to support ethical behavior [16]. In such circumstances, acting based on their personal moral framework may still foster a sense of moral comfort.

The results indicated that the mean score of moral courage among nurses working in ICUs was 83.66 ± 13.17 on a scale of 15–105. A high level of moral courage has also been reported in studies conducted in China [38], Finland [23], and Turkey [31], which aligns with the present study. The global aim of nursing is patient care, which includes principles such as nondiscriminatory treatment and the provision of high‐quality care. Accordingly, nurses should be committed to strengthening their moral courage and providing moral care, and managers should also be committed to morally supporting nurses to enhance their morally courageous behaviors [38].

In this study, the “moral goals” obtained the highest mean score, and the lowest mean score was related to “multiple values”, which is consistent with other studies [42, 43]. When taking action, a nurse must have the ability to apply various sets of values (organizational, personal, and environmental) to determine the right course of action [36]. Promoting multiple values in moral courage requires creating an environment in which diverse moral principles are recognized, reflected, or acted upon, even in challenging situations. Institutions can improve this dimension of moral courage by strengthening moral awareness, encouraging open communication, and providing support.

Another finding of the present study, which aligns with the findings of other studies, was that with increasing age, clinical experiences, and work experiences in ICU nurses, moral courage also increases [24, 29]. Generally, as age, work experiences, and familiarity with the work environment increase, nurses’ moral courage and their assertive behaviors also improve. Therefore, it seems that utilizing experienced nurses as role models for novice nurses can improve their moral courage behaviors.

The results of this study indicated that there is no relationship between moral comfort and demographic data. In the study by Abbasivand‐Jeyranha and Barkhordari‐Sharifabad, there was a significant relationship between nurses’ moral comfort and age, clinical work experience, work experiences in ICUs, marital status, education, and type of employment [14], which is inconsistent with the present study. This discrepancy may be due to contextual differences, such as variations in organizational structures, managerial support, peer support, open communication with the healthcare team, workloads, or the ethical climate of different units [20]. Differences in study populations may also contribute to these inconsistent findings, as individual factors such as self‐determination and accountability can influence moral comfort [20]. Further research is needed to clarify how these contextual factors influence the relationship between nurses’ demographic characteristics and moral comfort.

### 4.1. Limitations of the Study

One of the limitations of this research is the self‐reported nature of the data collection tool, which may have been influenced by participants’ social desirability, potentially affecting the accuracy of their responses. Although efforts were made to control this limitation by emphasizing the anonymity of the questionnaire and the confidentiality of the data, it may still have had an impact. In addition, the cross‐sectional nature of the study may limit the ability to draw conclusions about causality. Another limitation is the potential influence of unmeasured confounding variables that were not included in the analysis, which might have affected the observed associations between the study variables.

## 5. Conclusion

There was a significant positive correlation between moral courage and moral comfort, with moral courage predicting 35% of the variance in moral comfort. The findings indicate that moral courage is a significant predictor of moral comfort among ICU nurses. Nurses with higher levels of moral courage are more likely to experience moral comfort, which can enhance their psychological well‐being and professional performance in ethically challenging environments such as the ICU. These results underscore the critical role of moral courage in helping nurses navigate ethical dilemmas, cope with moral distress, and make decisions with greater moral confidence and comfort.

### 5.1. Implications for Nursing Practice

The findings of this study suggest that promoting moral courage can enhance nurses’ moral comfort. To achieve this, nursing managers and educators can implement targeted strategies. Structured training programs and ethical workshops help nurses recognize ethical challenges, make confident decisions, and strengthen their moral courage. Additionally, mentorship, supportive peer interactions, and open discussions about ethical dilemmas reinforce morally responsible behaviors. Furthermore, supportive leadership, clear ethical policies, and a fair, empowering work environment further facilitate nurses’ moral courage. By integrating these approaches, nurses are better equipped to navigate complex clinical situations, reduce moral distress, and provide high‐quality, ethically sound patient care.

## Disclosure

All authors read and approved the final manuscript.

## Conflicts of Interest

The authors declare no conflicts of interest.

## Author Contributions

All authors have participated in the conception and design of the study. Sara Etemadi and Parvin Babaei contributed to the data collection and prepared the first draft of the manuscript. Maasoumeh Barkhordari‐Sharifabad critically revised and checked closely the proposal, the analysis and interpretation of the data, and design the article. Mansoureh Zagheri‐Tafreshi carried out the analysis, interpretation of the data, and drafting the manuscript.

## Funding

No funding was received for this manuscript.

## Data Availability

The data that support the findings of this study are available on request from the corresponding author. The data are not publicly available due to privacy or ethical restrictions.
